# Therapeutic neutralizing monoclonal antibody administration protects against lethal yellow fever virus infection

**DOI:** 10.1126/scitranslmed.ade5795

**Published:** 2023-03-29

**Authors:** Michael J. Ricciardi, Lauren N. Rust, Nuria Pedreño-Lopez, Sofiya Yusova, Sreya Biswas, Gabriela M. Webb, Lucas Gonzalez-Nieto, Thomas B. Voigt, Johan J. Louw, Fernanda D. Laurino, John R. DiBello, Hans-Peter Raué, Aaron M. Barber-Axthelm, Kimberly Chun, Samantha Uttke, Lidiane M. S. Raphael, Aaron Yrizarry-Medina, Brandon C. Rosen, Rebecca Agnor, Lina Gao, Caralyn Labriola, Michael Axthelm, Jeremy Smedley, Justin G. Julander, Myrna C. Bonaldo, Laura M. Walker, Ilhem Messaoudi, Mark K. Slifka, Dennis R. Burton, Esper G. Kallas, Jonah B. Sacha, David I. Watkins, Benjamin J. Burwitz

**Affiliations:** 1 Mabloc LLC, 725 21st St. NW, Suite 301, Washington, DC 20052, USA.; 2 George Washington University, 2121 I St. NW, Washington, DC 20052, USA.; 3 Vaccine and Gene Therapy Institute, Oregon Health and Science University, Beaverton, OR 97006, USA.; 4 IrsiCaixa AIDS Research Institute, Ctra. del Canyet SN, Badalona 08916, Barcelona, Spain.; 5 Miller School of Medicine, University of Miami, Miami, FL 33136, USA.; 6 Oregon National Primate Research Center, Oregon Health and Science University, Beaverton, OR 97006, USA.; 7 Laboratório de Biologia Molecular de Flavivírus, Instituto Oswaldo Cruz, Fundação Oswaldo Cruz, Rio de Janeiro, Brazil.; 8 Institute for Antiviral Research, Utah State University, Logan, UT 84322, USA.; 9 Adagio Therapeutics Inc., Waltham, MA 02451, USA.; 10 Department of Microbiology, Immunology, and Molecular Genetics, University of Kentucky, Lexington, KY 40536, USA.; 11 Department of Immunology and Microbiology, Scripps Research Institute, La Jolla, CA 92037, USA.; 12 Department of Infectious and Parasitic Diseases, School of Medicine, University of Sao Paulo, Sao Paulo, Brazil.

## Abstract

Yellow fever virus (YFV) is a reemerging global health threat, driven by several factors, including increased spread of the mosquito vector and rapid urbanization. Although a prophylactic vaccine exists, vaccine hesitancy, supply deficits, and distribution difficulties leave specific populations at risk of severe YFV disease, as evidenced by recent outbreaks in South America. To establish a treatment for patients with severe YFV infection, we tested 37 YFV-specific monoclonal antibodies isolated from vaccinated humans and identified two capable of potently neutralizing multiple pathogenic primary YFV isolates. Using both hamster and nonhuman primate models of lethal YFV infection, we demonstrate that a single administration of either of these two potently neutralizing antibodies during acute infection fully controlled viremia and prevented severe disease and death in treated animals. Given the potential severity of YFV-induced disease, our results show that these antibodies could be effective in saving lives and fill a much-needed void in managing YFV cases during outbreaks.

## INTRODUCTION

Yellow fever virus (YFV) infection results in high viral loads and a mortality rate of up to 50% of hospitalized patients (1). Even with the availability of the live-attenuated YFV-17D vaccine, recently published estimates indicate an annual incidence of 109,000 severe YFV cases, with 51,000 related deaths (2). Despite numerous vaccine campaigns, immunization coverage remains low in some vulnerable populations, resulting in a notable number of at-risk individuals, with the potential for international spread (3). This is exacerbated by the uncommon (1 in 250,000) but severe side effects caused by the YFV-17D vaccine, which can lead to severe illness, organ failure, or death (4, 5). During the 2016–2019 outbreak in Brazil, the World Health Organization reported about 100 cases of severe adverse events due to mass vaccination campaigns, which dissuaded many people from receiving the vaccine (3).

There is currently no approved treatment for YFV infection. Although there have been numerous other monoclonal antibodies (mAb) isolated from previously infected humans, vaccinated individuals, and various sources, there are now no active clinical trials testing interventional YFV therapeutics (6–10). Previous clinical trials have included two limited studies. A phase 1b clinical trial (NCT03891420), which compared intravenous infusion of the adenosine nucleoside analog galidesivir against placebo in patients hospitalized for YFV infection, was terminated by the sponsor before full enrollment. Another phase 1a/b clinical trial (NCT03776786) studied the safety, pharmacokinetics, and efficacy of an intravenously administered human mAb TY104. The sponsors found that the mAb infusions were well tolerated and efficacious; however, the study faced data limitations, only measuring abrogation of viremia after vaccination with YFV-17D, an attenuated YFV vaccine strain (11). Thus, no clinical data exist showing successful drug interventions for severe YFV infection.

We recently published over 1200 YFV-specific mAb sequences from YFV-17D–vaccinated volunteers (12). However, these antibodies were not characterized for neutralization against primary YFV strains nor tested in vivo. Here, we characterize a subset of these human IgG1 mAbs for their ability to neutralize laboratory-adapted and primary isolates of YFV in vitro and suppress YFV replication in vivo. We show that several mAbs successfully neutralize primary isolates of YFV obtained from patients in Brazil at half-maximal inhibitory concentration (IC_50_) values of <50 ng/ml. We also show that passive immunization with these potently neutralizing YFV-specific mAbs can ameliorate YFV disease in both Syrian golden hamsters and Indian rhesus macaques (RMs), providing a strong rationale for expedited clinical development of this intervention.

## RESULTS

### Screening and selection of YFV-neutralizing mAbs

**Fig. 1.**
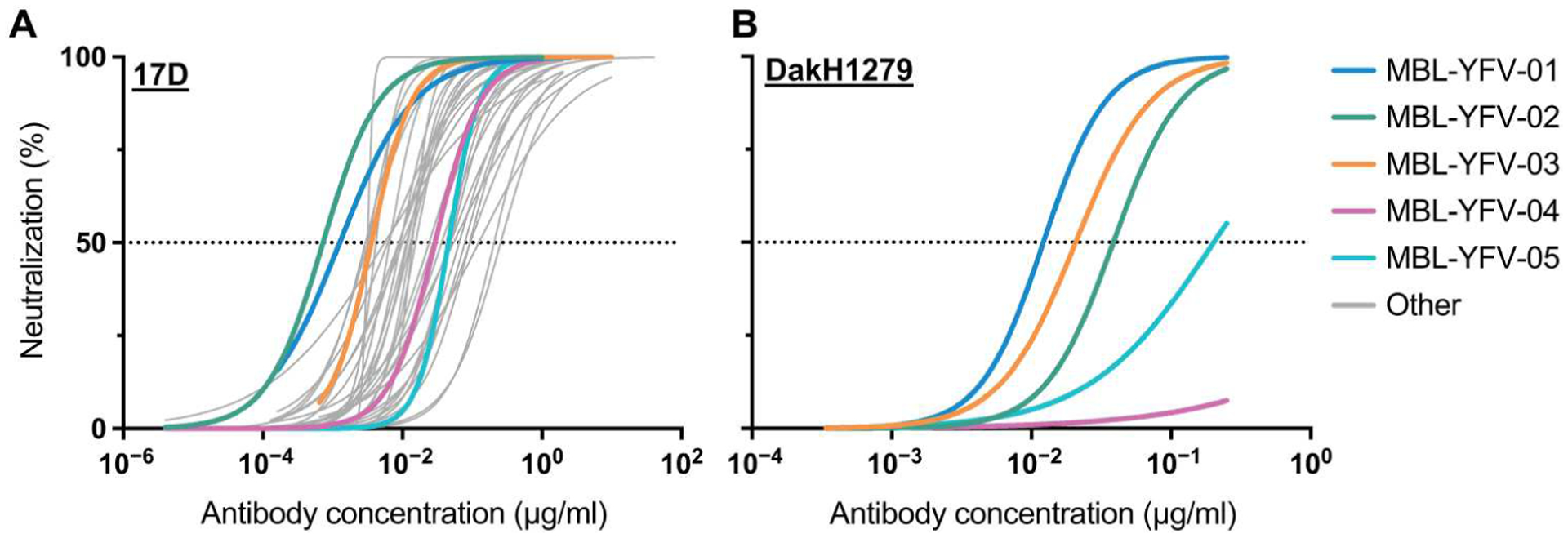
Screening and selection of YFV-neutralizing mAbs. (**A**) Thirty-seven mAbs were tested for neutralization of YFV-17D in a focus-forming assay, and IC_50_ was calculated. (**B**) Five mAbs were tested for neutralization of YFV-DakH1279 in a focus-forming assay, and IC_50_ was calculated.

We screened 37 YFV-reactive mAbs, isolated from memory B cells of YFV-17D–immunized volunteers (12), for neutralization against YFV-17D (Fig. 1A and fig. S1, A and B). Twenty-nine of these mAbs targeted YFV envelope (E) domain II (DII), four targeted YFV E DIII, and four others were unable to be clearly mapped (Table 1). All DII-reactive mAbs outcompeted another previously isolated mAb, 5A, which binds a DII fusion loop proximal epitope and showed protection in a mouse YFV challenge study (13). From this initial analysis, we selected five mAbs (MBL-YFV-01 through MBL-YFV-05) for further testing on the basis of three main criteria: (i) neutralization of YFV-17D with an IC_50_ < 50 ng/ml, (ii) neutralization of a primary wild-type isolate (YFV-ES504; South America genotype I) from the 2016–2017 outbreak in Brazil (14) with an IC_50_ < 31 ng/ml, and (iii) complete neutralization of YFV-ES504 with a minimum mAb concentration to reduce viral plaques by 100% (*V*_max_) ≤ 500 ng/ml. One of these five mAbs also showed binding to other tested flaviviruses, including Zika, dengue, and West Nile virus (Table 1). Neutralization synergy studies were also performed with these five mAbs, but no synergy was observed.

Next, we performed in vitro escape assays, using a previously published serial flavivirus passage method (15, 16), to assess mAb-driven viral mutations emerging in the parental YFV-17D infection strain. MBL-YFV-01, MBL-YFV-02, MBL-YFV-03, and MBL-YFV-05 did not generate any YFV-17D-E nucleotide or amino acid mutations at any point throughout 10 passages using different concentrations of the mAbs. In contrast, MBL-YFV-04 drove the emergence of several point mutations in YFV-17D-E across all three domains (table S1).

In preparation for in vivo testing of these mAbs, we assessed the ability of these five mAbs to neutralize the pathogenic YFV strain DakH1279 (West Africa genotype II), an isolate from a YFV-infected patient in Senegal in 1965 that has been used previously in nonhuman primate challenge studies (Fig. 1B and fig. S1, C and D) (17). On the basis of these measurements, we selected two antibodies (MBL-YFV-01 and MBL-YFV-02) that did not compete with each other [one DII-specific and one DIII-specific as outlined in previous studies (12)], neutralized YFV-DakH1279 with an IC_50_ < 50 ng/ml, and completely neutralized YFV-ES504 with a *V*_max_ of ≤250 ng/ml. These antibodies neutralized YFV-17D better than YFV-DakH1279, although this is most likely due to YFV-17D being used as the immunogen to derive the antibodies in patients.

Last, we assessed the capacity of these two mAbs to neutralize three additional primary isolates from Brazil (YFV-4408–1E, YFV-RJ155, and YFV-GO09; all South America genotype I subclade 1E) (Table 2). These strains were chosen because they were isolated during different outbreaks (18). YFV-4408–1E was isolated from a YFV-infected howler monkey in 2008 in Rio Grande do Sul state. YFV-RJ155, similar to the YFV-ES504 strain, was obtained from the most recent outbreak in southeastern Brazil (2016–2017). YFV-GO09 was isolated in Goiás (2017), yet it was not related to the 2016–2017 outbreak. Both mAbs had an IC_50_ < 15 ng/ml against all of these primary, highly pathogenic isolates.

### Therapeutic mAb administration protects hamsters from pathogenic YFV infection

**Fig. 2.**
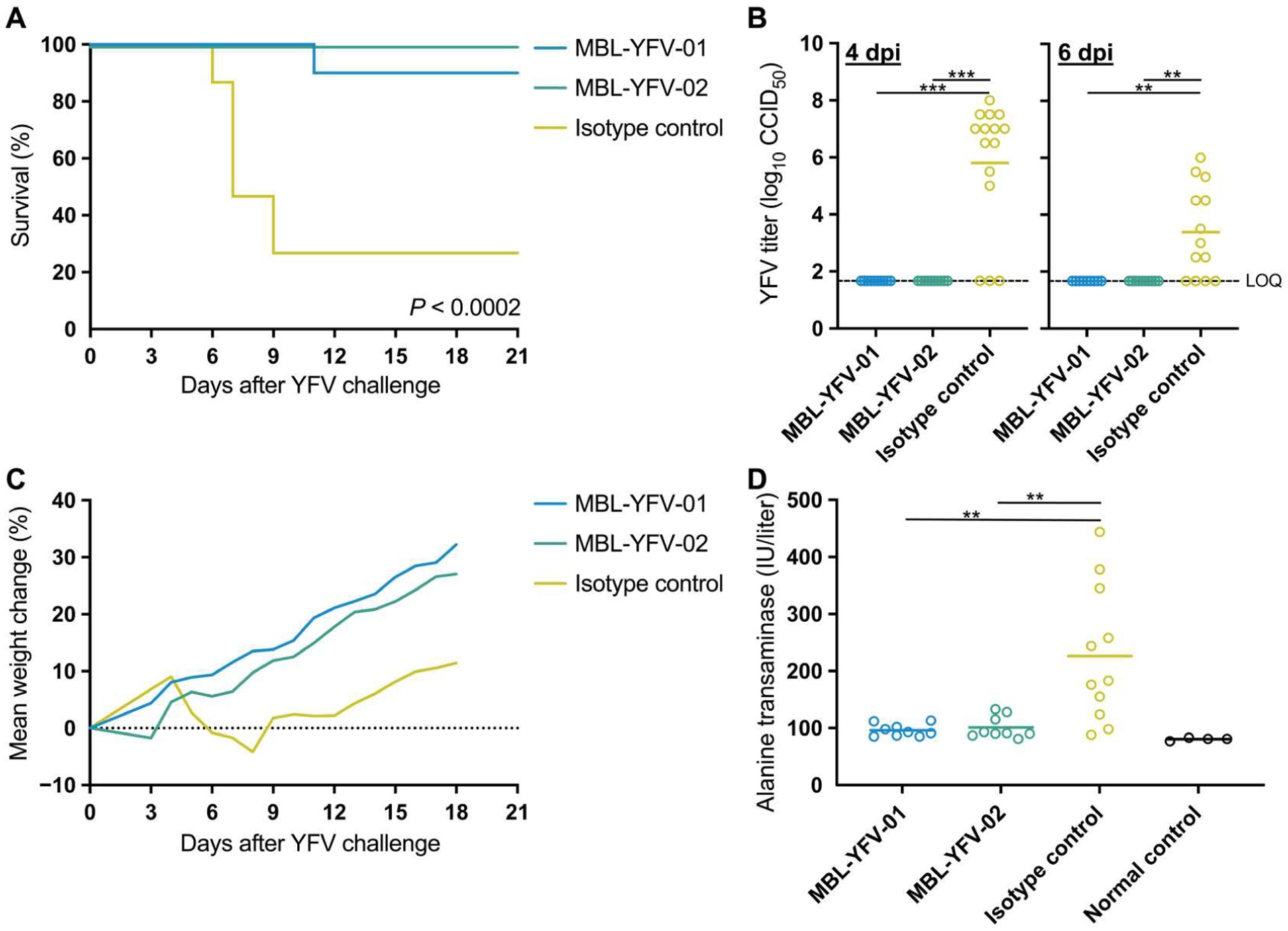
Therapeutic mAb administration protects hamsters from pathogenic YFV infection. (**A**) Kaplan-Meier survival curves of Syrian golden hamsters after challenge with YFV-Jimenez and treatment with YFV-specific or isotype control antibodies. *P* value determined by Mantel-Cox test with Bonferroni correction. (**B**) YFV-Jimenez titers in the sera of Syrian golden hamsters at 4 and 6 dpi as measured by an ex vivo infection assay to determine CCID_50_. One hamster from the MBL-YFV-01 group had insufficient serum at 6 dpi to measure YFV-Jimenez titers. *P* values determined by Mann-Whitney test with Bonferroni correction. Limit of quantification (LOQ), 1.67 log_10_. (**C**) Longitudinal mean weight change of each hamster treatment group after challenge with YFV-Jimenez. (**D**) Serum ALT levels of each hamster 6 dpi and measurements from normal controls. Two hamsters in the isotype control group and one hamster in the MBL-YFV-01 group had insufficient serum to measure ALT levels. *P* values determined by Mann-Whitney test with Bonferroni correction. **P < 0.01 and ***P < 0.001.

Having shown that the two candidate mAbs neutralize pathogenic lab strains and primary isolates of YFV at IC_50_ < 50 ng/ml, we next designed in vivo experiments to test the efficacy of mAb administration during acute YFV infection. Syrian golden hamsters were infected with 200 cell culture infectious doses (CCID_50_) of the serially passaged, hamster-adapted YFV-Jimenez strain. Three days post infection (dpi), we administered a single treatment of either one of our mAb candidates or an SIV-specific, isotype-matched control intraperitoneally at a dose of 20 mg/kg. We monitored disease parameters including survival, viremia, weight change, and serum alanine transaminase (ALT) levels to evaluate the efficacy of the mAb treatments. Both YFV-specific mAbs significantly increased survival, with MBL-YFV-02 administration leading to survival in nine of the nine animals (Fig. 2A; *P* < 0.0002). In contrast, 11 of the 15 hamsters treated with the SIV-specific, isotype-matched mAb required euthanasia because of severe disease. Next, we assessed YFV infectious titers in the sera of hamsters using an ex vivo infectivity assay. We found that only hamsters treated with SIV-specific, isotype-matched mAb had replication-competent YFV in their sera (Fig. 2B). We also measured weights daily and found that hamsters receiving SIV-specific mAb treatment showed significantly reduced weight gain beginning at 6 dpi (Fig. 2C and table S2). The four isotype control hamsters that survived past 9 dpi also began to gain weight after 12 dpi (Fig. 2C). Last, we used serum ALT measurements as a biomarker of liver disease. We found significantly elevated ALT at 6 dpi only in hamsters with replication-competent YFV in their sera, whereas YFV-specific mAb–treated hamsters exhibited ALT levels similar to those of normal hamsters (Fig. 2D; *P* < 0.01). These data show that YFV-specific mAb administration to hamsters after pathogenic YFV challenge can completely reverse disease course and protect hamsters from death.

### Therapeutic mAb administration protects RMs from pathogenic YFV infection

**Fig. 3.**
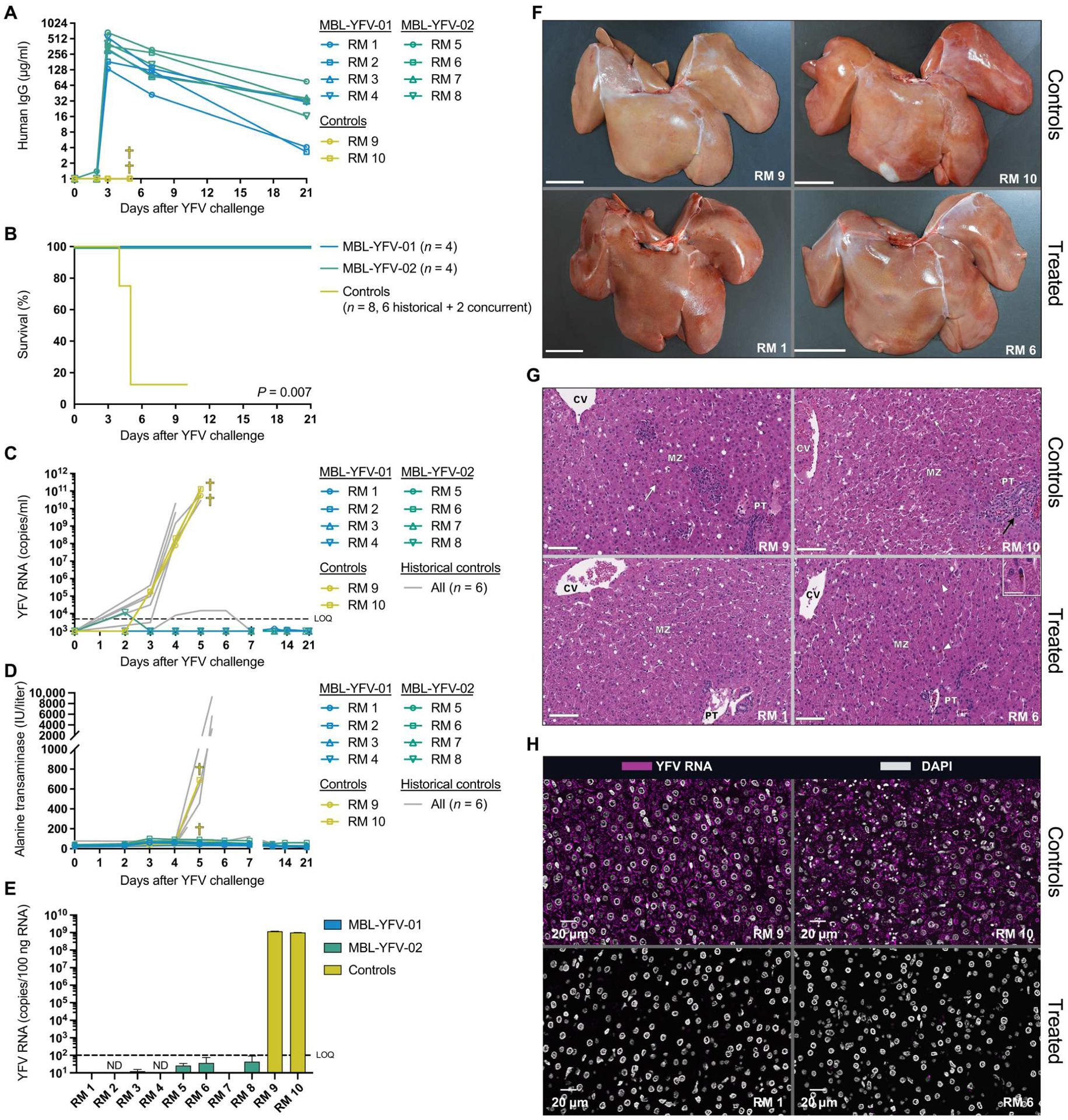
Therapeutic mAb administration protects RM from pathogenic YFV infection. (**A**) Longitudinal concentration of MBL-YFV-01 and MBL-YFV-02 in the plasma of YFV-DakH1279 challenged RMs. (**B**) Kaplan-Meier survival curves of RMs after challenge with YFV-DakH1279 and treatment with YFV-specific antibodies. *P* value determined by Mantel-Cox test with Bonferroni correction. (**C**) Longitudinal serum YFV-DakH1279 loads in RMs, including historical controls. LOQ, 5 × 10^3^ copies per ml. (**D**) Longitudinal serum ALT levels in RMs. (**E**) Quantification of YFV-DakH1279 RNA in the livers of RMs at necropsy. LOQ, 1 × 10^2^ copies per 100 ng of RNA. (**F**) Gross pathology of livers from YFV-DakH1279–infected RMs. Scale bars, 3 cm. (**G**) Hematoxylin and eosin staining of livers from YFV-DakH1279–infected RMs displaying midzonal necrosis with Councilman bodies (white arrow) in the control animals and comparably normal livers with minimal midzonal mononuclear cell infiltrates (black arrowhead) or rare midzonal Kupffer cells containing intracytoplasmic golden-tan pigment (white arrowheads; inset: scale bar, 25 μm) in the mAb-treated animals. Scale bars, 100 μm. PT, portal triad; MZ, midzonal region; CV, central vein. (**H**) RNAScope staining of YFV-DakH1279 RNA in the livers of YFV-DakH1279–infected RMs. DAPI, 4′,6-diamidino-2-phenylindole.

Given the success of YFV-specific mAb treatment in hamsters, we next tested MBL-YFV-01 and MBL-YFV-02 in a relevant, preclinical, nonhuman primate model of pathogenic YFV infection. We infected 10 RMs (table S3) with 1 × 10^3^ CCID_50_ of YFV-DakH1279, a YFV inoculum previously shown to lead to clinical endpoints requiring euthanasia in most RMs by 5 dpi (17). Two days after YFV infection, we intravenously administered either MBL-YFV-01 or MBL-YFV-02 (50 mg/kg) to eight RMs (*n* = 4 per group) and monitored concentrations in the plasma. mAb levels reached a mean concentration of 368 μg/ml and were detectable through euthanasia at 21 dpi (Fig. 3A). Two RMs served as concurrent, untreated controls, and we included six historical control RMs from our former study, because they were infected with the same cryopreserved stock, dose, and route of YFV-DakH1279 (17). All mAb-treated animals survived through 21 dpi, whereas both concurrent control animals required euthanasia by 5 dpi. Thus, in line with previous studies of this dose, seven of the eight control RMs required euthanasia (Fig. 3B) (17). We found a significant increase in survival of mAb-treated RMs compared with controls (*P* = 0.007), and this significance was maintained when historical controls were removed from the analysis (*P* = 0.0111). Animals treated with mAb had no detectable YFV in the serum, with the exception of RM 8 at 2 dpi (Fig. 3C). In contrast, both control RMs had viral loads above 1 × 10^10^ copies per ml at the time of euthanasia. Two-way repeated measures analysis of variance (ANOVA) including the historical controls showed significant reductions in viral loads beginning at 3 dpi (table S2). Next, serum ALT measurements were used as a biomarker of liver dysfunction. In animals treated with MBL-YFV-01 or MBL-YFV-02, we saw a transient two- and fourfold mean ALT increase at 3 dpi, respectively, when compared with baseline (Fig. 3D). A 22-fold increase in peak ALT level was seen in RM 10, whereas RM 9 had only a modest threefold rise before meeting other criteria for euthanasia (Fig. 3D). YFV RNA was either undetectable or below the level of quantification in the livers of mAb-treated animals but reached high levels in the control RMs (Fig. 3E). Supporting these findings, treated animals had no gross liver pathology (Fig. 3F and fig. S2) and, on histology, had minimal changes. These included sparse midzonal aggregates of small numbers of mononuclear cells occasionally associated with single or few degenerate hepatocytes and scant midzonal Kupffer cells with intracytoplasmic golden-tan pigment, suggestive of Villela bodies (Fig. 3G and fig. S3). Conversely, control animals had significant hepatic and nephrological necrosis at the time of euthanasia, which was reflected on histology (Fig. 3G and figs. S3 and S4). Additional pathological findings are summarized in the supplementary pathology report and table S4. Last, liver staining for YFV-E antigen and RNA revealed near-complete suppression of YFV in treated animals, although YFV RNA was rarely detected in the liver of RM 6 (Fig. 3H and figs. S5 and S6). In stark contrast, nearly all cells in control livers showed high levels of YFV RNA, highlighting the rapid and explosive viral replication kinetics of YFV in this model.

## DISCUSSION

There is an urgent need to develop a treatment strategy for YFV infection. In intensive care units, general medical support is the clinical standard (19). mAbs are increasingly being used to treat a variety of infectious diseases (20) and can even be effective against viruses that generate very high plasma viral concentrations and cause high mortality rates similar to that of YFV, such as Ebola virus (21–24). Multiple recent reports have shown complete protection from pathogenic YFV infection in mice and Syrian golden hamsters after infusion of a YFV-neutralizing mAb (9, 21). Here, we support these findings in Syrian golden hamsters and extend our understanding further by showing mAb protection in a highly pathogenic nonhuman primate YFV challenge model.

Our studies demonstrate that a single mAb delivered 2 days after YFV infection at 50 mg/kg was sufficient to prevent severe disease and death in nonhuman primates. The RM model closely resembles human disease progression after YFV infection, particularly in regard to liver dysfunction, hepatocyte necrosis, and the formation of hallmark councilman bodies (17). However, our study has limitations, including that YFV pathogenesis progresses more rapidly in hamsters and RMs compared with humans (17, 21, 25, 26), and, therefore, the optimal timing for treatment in humans is difficult to determine from our data. In addition, the number of RMs used in this study was small. Future studies using larger numbers of RMs and delayed mAb treatment could provide insight into the potential treatment window for patients in the clinic. However, such studies must carefully balance treatment timing with clinical readouts such as ALT and plasma viral loads, and given the extreme pathogenicity of YFV in macaques, such studies may not be feasible.

In terms of translation of our mAb therapy to the clinic, the ability to use a single mAb, rather than a cocktail of mAbs, markedly expedites the pathway to first-in-patient trials. Furthermore, single mAb therapies have recently been shown to be effective therapeutic agents against SARS-CoV-2 infections and have been widely accepted and tolerated in humans (27, 28).

YFV infection can lead to liver failure, with transplant often being the only available intervention. In the first 2 months of 2018, surgeons at the University of São Paulo performed five liver transplants in an attempt to save the lives of YFV-infected individuals (29, 30). The projected annualized costs in U.S. dollars for one liver transplant range from $1,427,805 to $2,093,789, which would require only about 10 cases to cover the initial costs of manufacturing one of these mAbs (31).

During the 2016 outbreak in Brazil, attempts to vaccinate individuals in the affected areas were often too late to prevent infection and disease. Several individuals who became infected and hospitalized with YFV had just received the vaccine (1). Generally, vaccine-induced IgM immunity can be seen within 10 days. However, in this specific outbreak, the time to induce protective neutralizing antibodies was delayed, particularly in the elderly (1, 32). Therefore, ring-fencing an epidemic by vaccination has limitations in controlling YFV outbreaks, and a readily available therapeutic could help those who fall ill and reduce transmission within these outbreak areas.

In most of North America and Europe, YFV disease is typically a direct result of vaccine-related adverse events that result in vaccine-associated viscerotropic or neurotropic disease caused by vaccine virus replication (33). In these instances, MBL-YFV-01 or MBL-YFV-02 could potentially be used to reduce vaccine-related viremia and thus curtail vaccine-related illness and limit vaccine hesitancy. Furthermore, most of North America and Europe still remain vulnerable to YFV infection, because these populations are generally unvaccinated. These primarily YFV-naïve areas could be primed for YFV outbreaks because there are examples of other flavivirus-induced diseases rapidly spreading, as shown by the recent emergence of dengue and Zika virus (34–36). Here, we show that MBL-YFV-01 and MBL-YFV-02 are effective against a variety of recently circulating YFV strains from South America, and these life-saving medicines could be developed rapidly as a safe and therapeutically efficacious drug for YFV infection and YFV vaccine–related illness.

## MATERIALS AND METHODS

### Study design

We designed this study to test our hypothesis that administration of YFV-neutralizing mAbs could rescue Syrian golden hamsters and RMs from severe disease and death after pathogenic YFV infection. Group sizes were selected to power survival analysis, given the highly lethal nature of YFV in both Syrian golden hamsters and RMs. Hamsters were randomly assigned to control and treatment groups. RMs were apportioned into groups to distribute the ages of monkeys. Study details, including YFV strains, doses, and routes for each challenge as well as treatments and measurements are defined in the Results and this section. Study endpoint was prospectively defined as 21 dpi for both hamsters and RMs on the basis of historical data. Authors were not blinded to treatment or animal group assignments. All animal studies were conducted after approval by the Utah State University and Oregon Health and Science University’s Institutional Animal Care and Use Committee using the standards of the National Institutes of Health (NIH)’s *Guide for the Care and Use of Laboratory Animals*.

### Viruses

The YFV-17D [American Type Culture Collection (ATCC) VR-1506] was prepared by propagating the virus in Vero cells (ATCC CCL-81) for two passages after virus isolation. YFV-DakH1279 (originally isolated from a YF patient in Senegal in 1965) was obtained from the World Reference Center for Emerging Viruses and Arboviruses after approval from R. Tesh (University of Texas Medical Branch, Galveston, TX). The YFV-DakH1279 challenge stock used in this study was previously titered and described (17). The isolation and characterization of the primary YFV isolates YFV-ES504, YFV-4408–1E, YFV-RJ155, and YFV-GO09 were previously described (18). For screening against DENV, the following strains were used: DENV1 (West Pac74; U88535.1), DENV2 (New Guinea C; AF038403.1), DENV3 (Sleman/78; AY648961), DENV4 (Dominica/8129; AF326573.1), and ZIKV (Paraiba/2015; KX280026), which were propagated in Vero cells. Briefly, after cytopathic effects were observed in Vero cells, the supernatant was harvested and centrifuged twice at 10,000 rpm for 15 min at 4°C to remove cell debris. The cleared viral supernatant was aliquoted, frozen at −80°C, and used for binding and neutralization assays. All stocks were quantified by viral plaque assay in Vero cells. West Nile virus cross-reactivity was determined by West Nile virus reporter viral particles previously described (12).

### Antibody expression and purification

Antibody heavy chain and light chain variable regions were cloned into human immunoglobulin G (IgG), human IgK, and human IgL backbone constructs. These were transiently transfected and expressed using the Expi293 Expression System (Thermo Fisher Scientific). After 6 days, culture supernatants were harvested, spun down, and sterile-filtered. mAbs were purified from supernatant using Protein A columns (Cytiva) and eluted under sterile conditions. Purified mAb used for in vivo experiments was then confirmed to be endotoxin-free (Pierce).

### Focus reduction neutralization tests

Focus reduction neutralization tests were conducted as previously described (37). Briefly, mAbs were serially diluted (7 × fivefold dilutions, each dilution in biological triplicate) in OptiMEM supplemented with 10% fetal bovine serum (FBS) and antibiotics. Virus was diluted to a final concentration of about 500 to 1000 plaque-forming units per ml in the same diluent and was added to equal volumes of the mAb-diluted sample and mixed. The virus/mAb mixture was incubated at 37°C for 1 hour. Cell culture medium was removed from 100% confluent monolayer cultures of Vero cells on 96-well plates, and 100 μl of the virus/mAb mixture were transferred onto triplicate cell monolayers. Cell monolayers were incubated for 1 hour at 37°C with the virus/mAb mixture and then overlaid with 1% methylcellulose in OptiMEM supplemented with 2% FBS, 2 mM glutamine, and gentamicin (50 μg/ml). Samples were incubated at 37°C for 2 days, after which plaques were visualized by immunoperoxidase staining, and an IC_50_ was calculated. Each experiment was performed twice for confirmation.

### In vitro escape assay

Vero cells were infected with YFV-17D in the presence or absence of mAb. YFV-17D was used at a final multiplicity of infection of about 0.3. Individual purified mAbs were added to the virus at three different concentrations (2, 1, and 0.5 μg/ml) to maximize the opportunity for the virus to select for escape mutants. Virus with antibody or virus and medium mixtures were incubated for 1 hour at 37°C. Cells were infected with these mixtures (1 hour) and then washed and incubated in medium with or without mAb supplementation. On the fourth day after infection, 50 μl of the cell-free supernatant were used to infect a new batch of cells, and mAb was readded to the cultures. This process was repeated 10 times. From each passage, the remainder of the supernatant was frozen in aliquots for viral RNA sequencing. To sequence the envelope from serum virus, we isolated viral RNA (Qiagen), synthesized complementary DNA, and sent for Sanger sequencing using YFV-E–specific primer sets (table S5).

### YFV challenges (hamster)

Thirty-five female Syrian golden hamsters (LVG/Lak strain) supplied by Charles River were used. Hamsters were block-randomized by weight to experimental groups and individually marked with ear tags. A challenge dose of 200 CCID_50_ of YFV (Jimenez hamster-adapted strain) per hamster was administered via bilateral intraperitoneal injections at a total volume of 200 μl. Group sizes were as follows: MBL-YFV-01 (*n* = 10), MBL-YFV-02 (*n* = 10), and isotype control (*n* = 15). One hamster in the MBL-YFV-02 group died 2 days after YFV-Jimenez infection for reasons unrelated to the study and was removed from statistical analyses.

### Infectious cell culture assay (hamster)

Virus titer was quantified using an infectious cell culture assay where a specific volume of serum was added to the first tube of a series of dilution tubes. Serial dilutions were made and added to Vero cells. Ten days later, cytopathic effect was used to identify the endpoint of infection. Four replicates were used to calculate the CCID_50_ per milliliter of serum.

### Serum ALT assay (hamster)

Serum was collected via ocular sinus bleed at 6 dpi. ALT (SGPT) reagent (Teco Diagnostics, Anaheim, CA) was used, and the protocol was altered for use in 96-well plates. Briefly, 50 μl of aminotransferase substrate were placed in each well of a 96-well plate, and 15 μl of sample were added at timed intervals. The samples were incubated at 37°C, after which 50 μl of color reagent were added to each sample and incubated for 10 min as above. A volume of 200 μl of color developer was next added to each well and incubated for 5 min. The plate was then read on a spectrophotometer, and ALT concentrations were determined per the manufacturer’s instructions.

### YFV challenges and passive antibody administration (RM)

A total of 10 Indian-origin RMs were challenged subcutaneously with 1 × 10^3^ CCID_50_ YFV-DakH1279 and assigned to three experimental groups: intravenous treatment with antibody MBL-YFV-01 (50 mg/kg; *n* = 4), MBL-YFV-02 (50 mg/kg; *n* = 4), or no-antibody controls (*n* = 2). Antibodies were infused intravenously at 2 dpi, whereas controls did not receive any infusions. Animals were cared for at the Oregon National Primate Research Center with the approval of the Oregon Health and Science University’s Institutional Animal Care and Use Committee using the standards of the NIH’s *Guide for the Care and Use of Laboratory Animals*.

### Quantification of delivered human IgG (RM)

Enzyme-linked immunosorbent assay was used to detect delivered human mAbs in plasma. Half-area 96-well costar assay plates (Corning) were coated overnight at 4°C with monkey cross-adsorbed goat anti-human IgG (2 μg/ml; SouthernBiotech, no. 2049–01) diluted in phosphate-buffered saline (PBS). Plates were then washed five times with PBS-T (PBS + 0.05% Tween 20) and subsequently blocked with blocking buffer (5% nonfat dry milk in PBS) for 1 hour at 37°C. Standards were prepared in naïve RM plasma at a concentration of 50 μg/ml. Heat-inactivated plasma and standards were then serially diluted in blocking buffer. Plates were washed with PBS-T five times, and diluted standards and plasma samples were added to the designated wells. After an hour of incubation at 37°C, the plate was washed five times with PBS-T, and detection was carried out with goat anti-human IgG–horseradish peroxidase (SouthernBiotech, 2045–05) at a 1:10,000 dilution in blocking buffer for 1 hour at 37°C. Plates were then washed five times with PBS-T and developed for 1 min at room temperature using 3,3′,5,5′-tetramethylbenzidine substrate (SouthernBiotech). Reactions were stopped with 2 M HCl. Plates were read on the Synergy HTX Multi-Mode Microplate Reader (BioTek), and data were collected using software Gen5 v3.09 at two absorbance wave-lengths: 650 and 450 nm. The final optical density (OD) was determined by subtracting OD_650 nm_ from OD_450 nm_.

### YFV-DakH1279 RNA quantification (RM)

Viral RNA was extracted from 200 μl of serum using a QiaAmp MinElute Virus Spin kit (Qiagen, catalog no. 57704) according the manufacturer’s instructions. Total intracellular DNA and RNA were extracted from the liver tissues. Briefly, samples were snap-frozen in lysing matrix tubes (MP Bio, catalog no. 116913050-CF) soaked in 1 ml of TRI reagent (Molecular Research Center, catalog no. RN190) and homogenized at 4000 rpm for 1 min in a BeadBug microtube homogenizer (MilliporeSigma, catalog no. Z763713). First, RNA extraction was performed by addition of 1/10th volume bromochloropropane (Sigma-Aldrich, catalog no. B9673) to the TRI reagent, vortexing, incubating at room temperature for 5 min, and then spinning at 12,000*g* for 5 min to achieve phase separation. Glycogen (12 μl; Thermo Fisher Scientific, catalog no. 10814010) was added to a sterile 1.5-ml snap-cap tube, and the RNA-containing upper aqueous phase was added and placed on ice. Samples for DNA extraction were processed by addition of back extraction buffer [9.09 g of Tris base (Thermo Fisher Scientific, catalog no. 77-86-1), 3.75 ml of 1 M sodium citrate (Thermo Fisher Scientific, catalog no. BP327–500) solution, 50 ml of 6 M guanidine thiocyanate (Thermo Fisher Scientific, catalog no. AM9422), and brought to 75 ml with distilled water] to the sample tubes containing the remaining interphase/organic phase mix after RNA extraction. Samples were vortexed, incubated for 5 min at room temperature, and spun at 12,000*g* for 5 min to achieve phase separation followed by addition. Glycogen (12 μl; Thermo Fisher Scientific, catalog no. 10814010) was added to a sterile 1.5-ml snap-cap tube, and the DNA-containing upper aqueous phase was added and placed on ice. Next, both the DNA and RNA samples were treated with isopropanol (Thermo Fisher Scientific, catalog no. 383920025), mixed gently by inversion, and spun at 15,000*g* for 5 min at room temperature. The isopropanol was carefully aspirated, and 75% ethanol was added to the pellets. The tubes were again spun at 15,000*g* for 5 min, and the ethanol wash was repeated a second time. All residual ethanol was removed with a micropipette. The pellets were dried at 37°C on a heat block, followed by resuspending the pellets in 100 μl of Tris-EDTA buffer (Thermo Fisher Scientific, catalog no. 12090015). Tubes were placed back in the 37°C heat block and were shaken at 500 rpm for 15 min. Tubes were gently vortexed and placed on ice for use in assays. Quantity and integrity of the extracted DNA and RNA were assessed on the NanoDrop 2000 Spectrophotometer (Nanodrop Technologies, catalog no. ND2000).

YFV RNA from both serum and liver was quantified using the TaqPath 1-Step RT-qPCR Master Mix (Thermo Fisher Scientific, catalog no. A15299) using primers/probe: YFV_qPCR-F (5′-CACGGGTGTGACAGACTGAAGA-3′), YFV_qPCR-R (5′-CCAGGCCGAACCTGTCAT-3′), and YFV_qPCR-Probe (5′−6FAM-ATGGCGGTG/ZEN/AGTGGAGACGATTG-TAMRA-3′)using an annealing temperature of 60°C. All thermocycling parameters followed the manufacturer’s instructions. All thermocycling and quantification analyses were conducted on an QuantStudio 3 (Applied Biosystems, catalog no. A28567). Quantification was assessed relative to an absolute standard curve using synthesized RNA corresponding to the quantitative polymerase chain reaction target region.

### Serum ALT assay (RM)

Blood was collected into nonanticoagulant, clot activator tubes and spun down at 1860*g* for 10 min to separate the serum from the clotted blood. Serum (100 μl) was assessed by the Abaxis Vetscan VS2 Chemistry Analyzer for the quantitative analysis of ALT.

### Pathology assessment (RM)

Blood, liver, and kidney were collected upon euthanasia. Tissues were fixed in 4% paraformaldehyde for 24 hours and then placed in 70% ethanol at 4°C for 4 to 6 days before paraffin embedding. Sections were cut at 5 μm and stained with hematoxylin and eosin on the Leica ST5020 Autostainer, and whole slide scanning was performed by a Leica AT2 slide scanner at ×20 magnification. Slides were reviewed by two veterinary pathologists using HALO Link software (Indica Labs) and Leica DM 3000 LED microscopes.

### YFV RNA in situ hybridization (RM)

RNA detection was performed using the RNAscope 2.0 HD Multiplex detection protocol (ACD Life Sciences) followed by tyramide signal amplification (TSA; Invitrogen) according to the manufacturer’s protocol. Liver samples were fixed in 4% paraformaldehyde, processed for paraffin embedding, and cut at 7-μm thickness. Slides were heated at 60°C for 1 hour, dewaxed in xylenes for 10 min, and then placed in 100% ethanol for 5 min before air drying. Heat-induced epitope retrieval was performed by boiling sections in 1× RNAscope Pretreat 2 buffer [a citrate buffer (10 nM, pH 6); ACD Life Science, catalog no. 322000] for 30 min, immediately washed in deionized water, and then dehydrated in 100% ethanol for 5 min before air drying. Hydrophobic barrier pen was applied to encircle the section, and then the slides were incubated with RNAscope Protease III (ACD Life Sciences, catalog no. 322337) diluted 1:10 in PBS for 30 min at 40°C using a HybEZ hybridization oven (ACD Life Sciences). Slides were rinsed twice with deionized water and then incubated with 1.5% peroxide (Thermo Fisher Scientific, catalog no. H325–500) for 5 min at room temperature. Sections were again rinsed with deionized water and then incubated with prewarmed customized YFV RNAscope Probe (V-YFV-ENV-E; ACD Life Sciences, catalog no. 574391) in hybridization buffer A [6× SSC (1× SSC is 0.15 M NaCl, 0.015 M Na citrate), 25% formamide, 0.2% lithium dodecyl sulfate, blocking reagents] and incubated for 2 hours at 40°C. Slides were washed in wash buffer (0.1× or 0.05× SSC, 0.03% lithium dodecyl sulfate) and incubated with amplification reagents as described in the RNAscope 2.0 HD detection protocol (ACD Life Sciences, catalog no. 322310): amplifier 1 (2 nM) in hybridization buffer B (20% formamide, 5× SSC, 0.3% lithium dodecyl sulfate, 10% dextran sulfate, blocking reagents) at 40°C for 30 min; amplifier 2 (a proprietary enhancer to boost detection efficiency) at 40°C for 15 min; amplifier 3 (2 nM) in hybridization buffer B at 40°C for 30 min; amplifier 4 (2 nM) in hybridization buffer C (2× SSC, blocking reagents) at 40°C for 15 min; amplifier 5 (a proprietary signal amplifier) at room temperature for 30 min; and amplifier 6 (a proprietary secondary signal amplifier) at room temperature for 15 min. After each hybridization step, slides were washed with wash buffer three times at room temperature. Before detection, the slides were rinsed one time in 1× TBS Tween 20 (0.05% v/v). Amplifier 6 contained alkaline phosphatase (or horseradish peroxidase) labels, which were detected with an Alexa Fluor 488–conjugated TSA (Invitrogen, catalog no. B40953) solution for 10 min at room temperature. Slides were washed in TBS-T for 10 min and then stained with 4′,6-diamidino-2-phenylindole (5 mg/ml; Invitrogen, catalog no. D1306) diluted to 1:10,000 in TBS-T for 5 min at room temperature. Slides were washed, mounted with a SlowFade Gold anti-fade reagent (Invitrogen, catalog no. S36937), and cover-slipped. Images were acquired by scanning the entire tissue at ~40 Å magnification using the Olympus VS120 Slide Scanner and pseudo-colored using the Cell-Sens Dimension Desktop v1.18 software (Olympus).

### Repeated measures statistical analyses

Two-way repeated measures ANOVA was used for longitudinal outcome measures to compare yellow fever viral load (RMs) and mean weight change (hamsters) between the antibody-treated and control groups over the study period. In a typical experiment using repeated measures, two measurements taken several time points apart, optimal covariance structure chosen by Bayesian information criteria was used to account for within-subject correlation. Because of right-skewed data, the viral load analysis was performed on log-transformed data. Multiplicity-adjusted *P* values following Tukey procedures were presented. Statistical significance was determined at the significant α level of 0.05. Analyses were performed using SAS version 9.4, specifically PROC MIXED.

## Supplementary Material

Supp

Data file S1

MDAR Reproducibility Checklist

## Figures and Tables

**Table 1.** Summary of screened mAbs. N.B., nonbinding; Unk, unknown epitope. IC_50_, half-maximal inhibitory concentration of mAb to reduce viral plaques by 50%. *V*_max_, minimum mAb concentration to reduce viral plaques by 100%.

mAb ID	YFV-E domain	YFV-17D IC_50_ (ng/ml)	YFV-ES504 IC_50_ (ng/ml)	YFV-ES504 *V*_max_ (ng/ml)	Cross-reactivity	VH usage	VL usage
MBL-YFV-01	DII	1.3	<31	250	ZIKV, DV2, WNV	IGHV3-30	IGLV1-51
MBL-YFV-02	DIII	0.7	<31	<31	N.B.	IGHV3-33	IGLV1-44
MBL-YFV-03	DIII	3.6	<31	125	N.B.	IGHV3-33	IGLV1-44
MBL-YFV-04	DII	29.2	<31	500	N.B.	IGHV3-30	IGKV4-1
MBL-YFV-05	DII	45.7	<31	<31	N.B.	IGHV3-11	IGKV3D-15
MBL-YFV-06	DII	27.5	>1000	>1000	N.B.	IGHV4-4	IGLV1-51
MBL-YFV-07	DII	6.6	>1000	>1000	N.B.	IGHV3-33	IGKV1D-39
MBL-YFV-08	DII	3.2	>1000	>1000	N.B.	IGHV4-4	IGLV1-51
MBL-YFV-09	DII	36.5	>1000	>1000	N.B.	IGHV4-38-2	IGLV1-51
MBL-YFV-10	DII	64.9	<31	>1000	N.B.	IGHV4-4	IGLV1-51
MBL-YFV-11	DII	11.8	>1000	>1000	N.B.	IGHV4-4	IGLV1-51
MBL-YFV-12	DII	47.4	930	>1000	N.B.	IGHV4-4	IGLV1-51
MBL-YFV-13	DII	9.0	>1000	>1000	N.B.	IGHV4-4	IGLV1-51
MBL-YFV-14	DII	124.3	>1000	>1000	N.B.	IGHV4-61	IGKV3-11
MBL-YFV-15	DII	94.1	>1000	>1000	N.B.	IGHV3-11	IGLV3-21
MBL-YFV-16	DII	256.2	>1000	>1000	N.B.	IGHV3-11	IGLV1-51
MBL-YFV-17	DIII	49.3	340	1000	N.B.	IGHV3-23	IGKV1-33
MBL-YFV-18	DII	6.2	50	1000	N.B.	IGHV4-4	IGLV1-51
MBL-YFV-19	DII	10.1	>1000	>1000	N.B.	IGHV4-4	IGLV1-51
MBL-YFV-20	DIII	13.0	<31	1000	N.B.	IGHV3-23	IGKV3D-15
MBL-YFV-21	DII	205.9	>1000	>1000	N.B.	IGHV3-11	IGLV3-21
MBL-YFV-22	Unk	8.8	>1000	>1000	N.B.	IGHV3-30	IGLV3-21
MBL-YFV-23	Unk	3.2	<31	1000	N.B.	IGHV3-9	IGLV6-57
MBL-YFV-24	Unk	53.8	1000	>1000	N.B.	IGHV3-74	IGLV1-51
MBL-YFV-25	DII	48.6	>1000	>1000	N.B.	IGHV3-11	IGLV1-51
MBL-YFV-26	DII	24.0	>1000	>1000	N.B.	IGHV4-4	IGLV1-51
MBL-YFV-27	DII	13.3	>1000	>1000	N.B.	IGHV4-4	IGLV1-51
MBL-YFV-28	DII	4.2	40	>1000	N.B.	IGHV3-23	IGKV1-39/P
MBL-YFV-29	DII	3.2	1000	1000	N.B.	IGHV4-38-2	IGLV1-51
MBL-YFV-30	DII	9.4	>1000	>1000	N.B.	IGHV4-4	IGLV1-51
MBL-YFV-31	DII	3.0	1000	>1000	N.B.	IGHV3-11	IGLV2-14
MBL-YFV-32	DII	92.1	>1000	>1000	N.B.	IGHV3-11	IGLV2-14
MBL-YFV-33	DII	13.9	1000	>1000	N.B.	IGHV4-4	IGLV1-51
MBL-YFV-34	DII	5.6	>1000	>1000	N.B.	IGHV3-11	IGLV2-14
MBL-YFV-35	Unk	11.5	>1000	>1000	N.B.	IGHV3-30	IGKV1-33
MBL-YFV-36	DII	15.5	>1000	>1000	N.B.	IGHV4-61	IGKV3-11
MBL-YFV-37	DII	78.2	>1000	>1000	N.B.	IGHV3-30	IGLV2-14

**Table 2.** Selected mAbs for in vivo testing.

mAb ID	YFV-17D IC_50_ (ng/ml)	YFV-DakH1279 IC_50_ (ng/ml)	YFV-ES504 IC_50_ (ng/ml)	YFV-ES504 *V*_max_ (ng/ml)	YFV-4408-1E IC_50_ (ng/ml)	YFV-RJ155 IC_50_ (ng/ml)	YFV-GO09 IC_50_ (ng/ml)
MBL-YFV-01	1.3	12.2	<31	250	<15	<15	<15
MBL-YFV-02	0.7	38.6	<31	<31	<15	<15	<15

## Data Availability

All data associated with this study are present in the paper or the Supplementary Materials, with raw data available from the corresponding author upon request. mAbs are patented and available only with acceptable material transfer agreement.

## References

[1] Kallas EG, Zanella LDE, Moreira CHV, Buccheri R, Diniz GBF, Castiñeiras ACP, Costa PR, Dias JZC, Marmorato MP, Song ATW, Maestri A, Borges IC, Joelsons D, Cerqueira NB, Santiago ESNC, Claro IM, Sabino EC, Levi JE, Avelino-Silva VI, Ho Y-L, Predictors of mortality in patients with yellow fever: An observational cohort study. Lancet Infect. Dis 19, 750–758 (2019). 31104909 10.1016/S1473-3099(19)30125-2

[2] Gaythorpe KA, Hamlet A, Jean K, Garkauskas Ramos D, Cibrelus L, Garske T, Ferguson N, The global burden of yellow fever. eLife 10, e64670 (2021). 33722340 10.7554/eLife.64670 PMC7963473

[3] World Health Organization, Emergencies preparedness responses (2022); www.who.int/emergencies/disease-outbreak-news/item/09-March-2018-yellow-fever-brazil-en.

[4] Centers for Disease Control and Prevention, Yellow Fever Vaccine Information Statement (2011); www.cdc.gov/vaccines/hcp/vis/vis-statements/yf.html.

[5] Amanna IJ, Slifka MK, Questions regarding the safety and duration of immunity following live yellow fever vaccination. Expert Rev. Vaccines 15, 1519–1533 (2016). 27267203 10.1080/14760584.2016.1198259 PMC5171234

[6] Daffis S, Kontermann RE, Korimbocus J, Zeller H, Klenk HD, Ter Meulen J, Antibody responses against wild-type yellow fever virus and the 17D vaccine strain: Characterization with human monoclonal antibody fragments and neutralization escape variants. Virology 337, 262–272 (2005). 15919103 10.1016/j.virol.2005.04.031

[7] Julander JG, Thibodeaux BA, Morrey JD, Roehrig JT, Blair CD, Humanized monoclonal antibody 2C9-cIgG has enhanced efficacy for yellow fever prophylaxis and therapy in an immunocompetent animal model. Antiviral Res. 103, 32–38 (2014). 24393669 10.1016/j.antiviral.2013.12.011 PMC3951967

[8] Thibodeaux BA, Garbino NC, Liss NM, Piper J, Schlesinger JJ, Blair CD, Roehrig JT, A humanized IgG but not IgM antibody is effective in prophylaxis and therapy of yellow fever infection in an AG129/17D-204 peripheral challenge mouse model. Antiviral Res. 94, 1–8 (2012). 22366350 10.1016/j.antiviral.2012.02.001 PMC3331901

[9] Li Y, Chen Z, Wu L, Dai L, Qi J, Chai Y, Li S, Wang Q, Tong Z, Ma S, Duan X, Ren S, Song R, Liang M, Liu W, Yan J, Gao GF, A neutralizing-protective supersite of human monoclonal antibodies for yellow fever virus. Innovation (Camb) 3, 100323 (2022). 36199277 10.1016/j.xinn.2022.100323 PMC9529537

[10] National Institutes of Health, Clinical Trials Database (2022); clinicaltrials.gov.

[11] Low JG, Ng JHJ, Ong EZ, Kalimuddin S, Wijaya L, Chan YFZ, Ng DHL, Tan HC, Baglody A, Chionh YH, Lee DCP, Budigi Y, Sasisekharan R, Ooi EE, Phase 1 trial of a therapeutic anti-yellow fever virus human antibody. N. Engl. J. Med 383, 452–459 (2020). 32726531 10.1056/NEJMoa2000226

[12] Wec AZ, Haslwanter D, Abdiche YN, Shehata L, Pedreno-Lopez N, Moyer CL, Bornholdt ZA, Lilov A, Nett JH, Jangra RK, Brown M, Watkins DI, Ahlm C, Forsell MN, Rey FA, Barba-Spaeth G, Chandran K, Walker LM, Longitudinal dynamics of the human B cell response to the yellow fever 17D vaccine. Proc. Natl. Acad. Sci. U.S.A 117, 6675–6685 (2020). 32152119 10.1073/pnas.1921388117 PMC7104296

[13] Lu X, Xiao H, Li S, Pang X, Song J, Liu S, Cheng H, Li Y, Wang X, Huang C, Guo T, Meulen JT, Daffis S, Yan J, Dai L, Rao Z, Klenk HD, Qi J, Shi Y, Gao GF, Double lock of a human neutralizing and protective monoclonal antibody targeting the yellow fever virus envelope. Cell Rep. 26, 438–446.e5 (2019). 30625326 10.1016/j.celrep.2018.12.065

[14] Bonaldo MC, Gomez MM, Santos AAD, Abreu FVS, Ferreira-de-Brito A, Miranda RM, Castro MG, Lourenco-de-Oliveira R, Genome analysis of yellow fever virus of the ongoing outbreak in Brazil reveals polymorphisms. Mem. Inst. Oswaldo Cruz 112, 447–451 (2017). 28591405 10.1590/0074-02760170134 PMC5446234

[15] Sukupolvi-Petty S, Austin SK, Engle M, Brien JD, Dowd KA, Williams KL, Johnson S, Rico-Hesse R, Harris E, Pierson TC, Fremont DH, Diamond MS, Structure and function analysis of therapeutic monoclonal antibodies against dengue virus type 2. J. Virol 84, 9227–9239 (2010). 20592088 10.1128/JVI.01087-10 PMC2937608

[16] Magnani DM, Ricciardi MJ, Bailey VK, Gutman MJ, Pedreño-Lopez N, Silveira CGT, Maxwell HS, Domingues A, Gonzalez-Nieto L, Su Q, Newman RM, Pack M, Martins MA, Martinez-Navio JM, Fuchs SP, Rakasz EG, Allen TM, Whitehead SS, Burton DR, Gao G, Desrosiers RC, Kallas EG, Watkins DI, Dengue virus evades AAV-mediated neutralizing antibody prophylaxis in rhesus monkeys. Mol. Ther 25, 2323–2331 (2017). 28750738 10.1016/j.ymthe.2017.06.020 PMC5628771

[17] Engelmann F, Josset L, Girke T, Park B, Barron A, Dewane J, Hammarlund E, Lewis A, Axthelm MK, Slifka MK, Messaoudi I, Pathophysiologic and transcriptomic analyses of viscerotropic yellow fever in a rhesus macaque model. PLoS Negl. Trop. Dis 8, e3295 (2014). 25412185 10.1371/journal.pntd.0003295 PMC4238990

[18] Furtado ND, Raphael LM, Ribeiro IP, de Mello IS, Fernandes DR, Gomez MM, Dos Santos AAC, da Silva Nogueira M, de Castro MG, de Abreu FVS, Martins LC, da Costa Vasconcelos PF, Lourenco-de-Oliveira R, Bonaldo MC, Biological characterization of yellow fever viruses isolated from non-human primates in Brazil with distinct genomic landscapes. Front. Microbiol 13, 757084 (2022). 35237244 10.3389/fmicb.2022.757084 PMC8882863

[19] Kallas EG, Wilder-Smith A, Managing severe yellow fever in the intensive care: Lessons learnt from Brazil. J. Travel Med 26, taz043 (2019). 31180486 10.1093/jtm/taz043 PMC6621914

[20] Walker LM, Burton DR, Passive immunotherapy of viral infections: ‘Super-antibodies’ enter the fray. Nat. Rev. Immunol 18, 297–308 (2018). 29379211 10.1038/nri.2017.148 PMC5918154

[21] Doyle MP, Genualdi JR, Bailey AL, Kose N, Gainza C, Rodriguez J, Reeder KM, Nelson CA, Jethva PN, Sutton RE, Bombardi RG, Gross ML, Julander JG, Fremont DH, Diamond MS, Crowe JE Jr., Isolation of a potently neutralizing and protective human monoclonal antibody targeting yellow fever virus. mBio 13, e0051222 (2022). 35420472 10.1128/mbio.00512-22 PMC9239089

[22] Corti D, Misasi J, Mulangu S, Stanley DA, Kanekiyo M, Wollen S, Ploquin A, Doria-Rose NA, Staupe RP, Bailey M, Shi W, Choe M, Marcus H, Thompson EA, Cagigi A, Silacci C, Fernandez-Rodriguez B, Perez L, Sallusto F, Vanzetta F, Agatic G, Cameroni E, Kisalu N, Gordon I, Ledgerwood JE, Mascola JR, Graham BS, Muyembe-Tamfun JJ, Trefry JC, Lanzavecchia A, Sullivan NJ, Protective monotherapy against lethal Ebola virus infection by a potently neutralizing antibody. Science 351, 1339–1342 (2016). 26917593 10.1126/science.aad5224

[23] Mulangu S, Dodd LE, Davey RT, Mbaya OT, Proschan M, Mukadi D, Manzo ML, Nzolo D, Oloma AT, Ibanda A, Ali R, Coulibaly S, Levine AC, Grais R, Diaz J, Lane HC, Muyembe-Tamfum JJ; PALM Writing Group, Sivahera B, Camara M, Kojan R, Walker R, Dighero-Kemp B, Cao H, Mukumbayi P, Mbala-Kingebeni P, Ahuka S, Albert S, Bonnett T, Crozier I, Duvenhage M, Proffitt C, Teitelbaum M, Moench T, Aboulhab J, Barrett K, Cahill K, Cone K, Eckes R, Hensley L, Herpin B, Higgs E, Ledgerwood J, Pierson J, Smolskis M, Sow Y, Tierney J, Sivapalasingam S, Holman W, Gettinger N, Vallee D, Nordwall J; PALM Consortium Study Team, A randomized, controlled trial of Ebola virus disease therapeutics. N. Engl. J. Med 381, 2293–2303 (2019). 31774950 10.1056/NEJMoa1910993 PMC10680050

[24] Misasi J, Sullivan NJ, Immunotherapeutic strategies to target vulnerabilities in the Ebolavirus glycoprotein. Immunity 54, 412–436 (2021). 33691133 10.1016/j.immuni.2021.01.015

[25] Quaresma JAS, Pagliari C, Medeiros DBA, Duarte MIS, Vasconcelos PFC, Immunity and immune response, pathology and pathologic changes: Progress and challenges in the immunopathology of yellow fever. Rev. Med. Virol 23, 305–318 (2013). 23873723 10.1002/rmv.1752

[26] Avelino-Silva VI, Thomazella MV, Marmorato MP, Correia CA, Dias JZC, Maestri A, Cerqueira NB, Moreira CHV, Buccheri R, Felix AC, Zanella L, Costa PR, Kallas EG, Viral kinetics in sylvatic yellow fever cases. J. Infect. Dis , jiac435 (2022). 10.1093/infdis/jiac435 36316804

[27] Westendorf K, Zentelis S, Wang L, Foster D, Vaillancourt P, Wiggin M, Lovett E, van der Lee R, Hendle J, Pustilnik A, Sauder JM, Kraft L, Hwang Y, Siegel RW, Chen J, Heinz BA, Higgs RE, Kallewaard NL, Jepson K, Goya R, Smith MA, Collins DW, Pellacani D, Xiang P, de Puyraimond V, Ricicova M, Devorkin L, Pritchard C, O’Neill A, Dalal K, Panwar P, Dhupar H, Garces FA, Cohen CA, Dye JM, Huie KE, Badger CV, Kobasa D, Audet J, Freitas JJ, Hassanali S, Hughes I, Munoz L, Palma HC, Ramamurthy B, Cross RW, Geisbert TW, Menacherry V, Lokugamage K, Borisevich V, Lanz I, Anderson L, Sipahimalani P, Corbett KS, Yang ES, Zhang Y, Shi W, Zhou T, Choe M, Misasi J, Kwong PD, Sullivan NJ, Graham BS, Fernandez TL, Hansen CL, Falconer E, Mascola JR, Jones BE, Barnhart BC, LY-CoV1404 (bebtelovimab) potently neutralizes SARS-CoV-2 variants. Cell Rep. 39, 110812 (2022). 35568025 10.1016/j.celrep.2022.110812 PMC9035363

[28] Pinto D, Park YJ, Beltramello M, Walls AC, Tortorici MA, Bianchi S, Jaconi S, Culap K, Zatta F, Marco AD, Peter A, Guarino B, Spreafico R, Cameroni E, Case JB, Chen RE, Havenar-Daughton C, Snell G, Telenti A, Virgin HW, Lanzavecchia A, Diamond MS, Fink K, Veesler D, Corti D, Cross-neutralization of SARS-CoV-2 by a human monoclonal SARS-CoV antibody. Nature 583, 290–295 (2020). 32422645 10.1038/s41586-020-2349-y

[29] Duarte-Neto AN, Cunha MDP, Marcilio I, Song ATW, de Martino RB, Ho YL, Pour SZ, Dolhnikoff M, Saldiva PHN, Duarte MIS, Takakura CF, Lima FR, Tanigawa RY, D′A Iglezias S, Kanamura CT, Dos Santos ABG, Perondi B, Zanotto PMA, D’Albuquerque LAC, Alves VAF, Yellow fever and orthotopic liver transplantation: New insights from the autopsy room for an old but re-emerging disease. Histopathology 75, 638–648 (2019). 31087672 10.1111/his.13904

[30] Song ATW, Abdala E, de Martino RB, Malbouisson LMS, Tanigawa RY, Andrade GM, Ducatti L, Doi AM, Pinho JRR, Gomes-Gouvea MS, de Mello Malta F, Arantes RM Jr., Tonacio AC, Pinto LF, Haddad LBP, Santos VR, Pinheiro RSN, Nacif LS, Galvao FHF, Alves VAF, Andraus W, D’Albuquerque LAC, Liver transplantation for fulminant hepatitis attributed to yellow fever. Hepatology 69, 1349–1352 (2019). 30218577 10.1002/hep.30273

[31] Habka D, Mann D, Landes R, Soto-Gutierrez A, Future economics of liver transplantation: A 20-year cost modeling forecast and the prospect of bioengineering autologous liver grafts. PLOS ONE 10, e0131764 (2015). 26177505 10.1371/journal.pone.0131764 PMC4503760

[32] Staples JE, Barrett ADT, Wilder-Smith A, Hombach J, Review of data and knowledge gaps regarding yellow fever vaccine-induced immunity and duration of protection. npj Vaccines 5, 54 (2020). 32655896 10.1038/s41541-020-0205-6 PMC7338446

[33] Menezes Martins R, da Luz Fernandes Leal M, Homma A, Serious adverse events associated with yellow fever vaccine. Hum. Vaccin. Immunother 11, 2183–2187 (2015). 26090855 10.1080/21645515.2015.1022700 PMC4635904

[34] Centers for Disease Control and Prevention, Locally acquired dengue--Key West, Florida, 2009–2010. MMWR Morb. Mortal. Wkly. Rep 59, 577–581 (2010). 20489680

[35] Likos A, Griffin I, Bingham AM, Stanek D, Fischer M, White S, Hamilton J, Eisenstein L, Atrubin D, Mulay P, Scott B, Jenkins P, Fernandez D, Rico E, Gillis L, Jean R, Cone M, Blackmore C, McAllister J, Vasquez C, Rivera L, Philip C, Local mosquito-borne transmission of Zika virus—Miami-Dade and Broward Counties, Florida, June-August 2016. MMWR Morb. Mortal. Wkly Rep 65, 1032–1038 (2016). 27684886 10.15585/mmwr.mm6538e1

[36] McCarthy M, First US case of Zika virus infection is identified in Texas. BMJ 352, i212 (2016). 26762624 10.1136/bmj.i212

[37] Ricciardi MJ, Magnani DM, Grifoni A, Kwon YC, Gutman MJ, Grubaugh ND, Gangavarapu K, Sharkey M, Silveira CGT, Bailey VK, Pedreno-Lopez N, Gonzalez-Nieto L, Maxwell HS, Domingues A, Martins MA, Pham J, Weiskopf D, Altman J, Kallas EG, Andersen KG, Stevenson M, Lichtenberger P, Choe H, Whitehead SS, Sette A, Watkins DI, Ontogeny of the B- and T-cell response in a primary Zika virus infection of a dengue-naive individual during the 2016 outbreak in Miami, FL. PLOS Negl. Trop. Dis 11, e0006000 (2017). 29267278 10.1371/journal.pntd.0006000 PMC5755934

