# Supplementary material for: Therapeutic neutralizing monoclonal antibody administration protects against lethal yellow fever virus infection
Source: Sci Transl Med. Author manuscript; Available in PMC 2023 Oct 31. (PMC10617428; doi:10.1126/scitranslmed.ade5795)
Supplement: Supp [file NIHMS1909437-supplement-Supp.pdf]

Science Translational Medicine
AAAS

Supplementary Materials for

Therapeutic neutralizing monoclonal antibody administration protects against lethal yellow fever virus infection

Michael J. Ricciardi *et al.*

Corresponding author: Benjamin J. Burwitz, burwitz@ohsu.edu

Sci. Transl. Med. **15**, eade5795 (2023)
DOI: 10.1126/scitranslmed.ade5795

The PDF file includes:

Supplementary pathology report
Figs. S1 to S6
Tables S1 to S5

Other Supplementary Material for this manuscript includes the following:

Data file S1
MDAR Reproducibility Report

Supplementary pathology report.

Eight out of the ten animals, across all groups, had mild lymphocytosis with or without leukocytosis which preceded and persisted throughout the study. In addition to elevated ALT at the experimental endpoint, RM 10 had leukopenia, which is consistent with acute YFV infection.

On gross examination of the control animals at 5 dpi, relevant findings included hepatic necrosis and mild icterus of the renal pelvises in both, as well as dehydration, mild splenomegaly, and peripancreatic lymphadenomegaly in RM 9. Histologic evaluation of the liver of RM 10 revealed midzonal to massive hepatic necrosis with numerous hypereosinophilic apoptotic hepatocytes (Councilman bodies), vacuolar hepatocyte degeneration, rare multinucleated hepatocytes (up to 8 nuclei), and few midzonal Kupffer cells containing intracytoplasmic golden-tan pigment which may be reminiscent of Villela bodies. Other findings were mild acute renal tubular degeneration, moderate cortical lymphoid depletion in peripancreatic lymph nodes, and minimal lymphoid necrosis in splenic germinal centers. Control RM 9 displayed mild midzonal necrosis and lymphoplasmacytic hepatitis with some Councilman bodies in the liver. The kidneys had mild acute tubular degeneration and necrosis, bile casts, and mild interstitial lymphocytic nephritis (**Fig. S4**). Both control animals had scant hepatocytes with nuclei containing marginated chromatin and a glassy lightly eosinophilic inclusion body, consistent with Torres bodies.

At 21 dpi, all livers in the antibody-treated animals were grossly normal (**Fig. S2**) and other lesions were incidental, comprising hypertrophic cardiomyopathy in RM 5, lymphadenomegaly and typhlitis in RM 8, and jejunal lymphangiectasis in RM 7. Potentially relevant histologic changes in the antibody-treated animals were minimal and limited to the liver (**Fig. S3**). As described above, the most consistent finding was rare to few midzonal mononuclear cell aggregates (mostly 10-50 cells) which variably expanded sinusoids, surrounded one to few degenerate hepatocytes, and/or were admixed with small amounts of cellular debris and erythrocytes. RM 3 displayed a single 300 µm focus of midzonal mononuclear inflammation centered around necrotic and degenerate hepatocytes. Three animals (RM 1, RM 5, and RM 6) displayed scattered midzonal Kupffer cells which were plump and had intracytoplasmic golden-tan to light brown pigment, which may be suggestive of Villela bodies as described above. RM 1 and RM 8 had a few hepatocytes with up to 12 nuclei, indicative of regeneration.

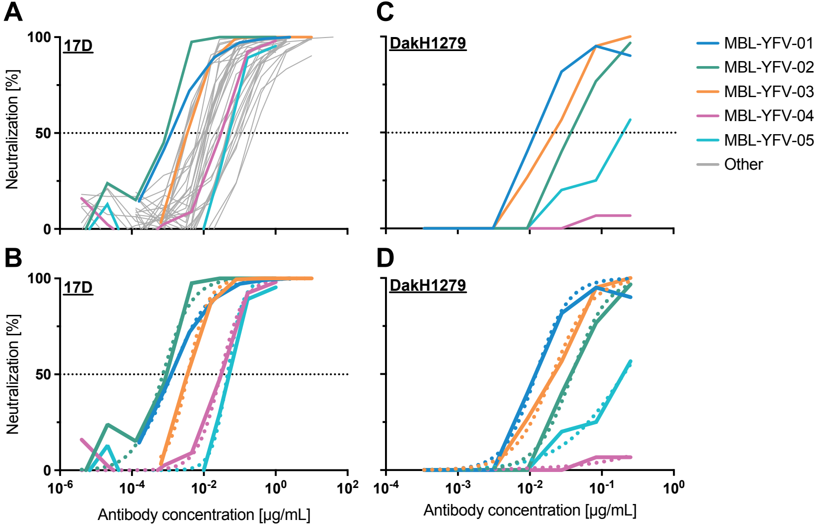

Figure S1. Screening and selection of YFV-neutralizing mAbs. A) Raw experimental data for 37 mAbs as represented in Fig. 1A. B) Overlay of raw experimental data (solid lines) and nonlinear regression curves (dotted lines) as represented in Fig. 1A. C) Raw experimental data for 5 mAbs as represented in Fig. 1B. D) Overlay of raw experimental data (solid lines) and nonlinear regression curves (dotted lines) as represented in Fig. 1B.

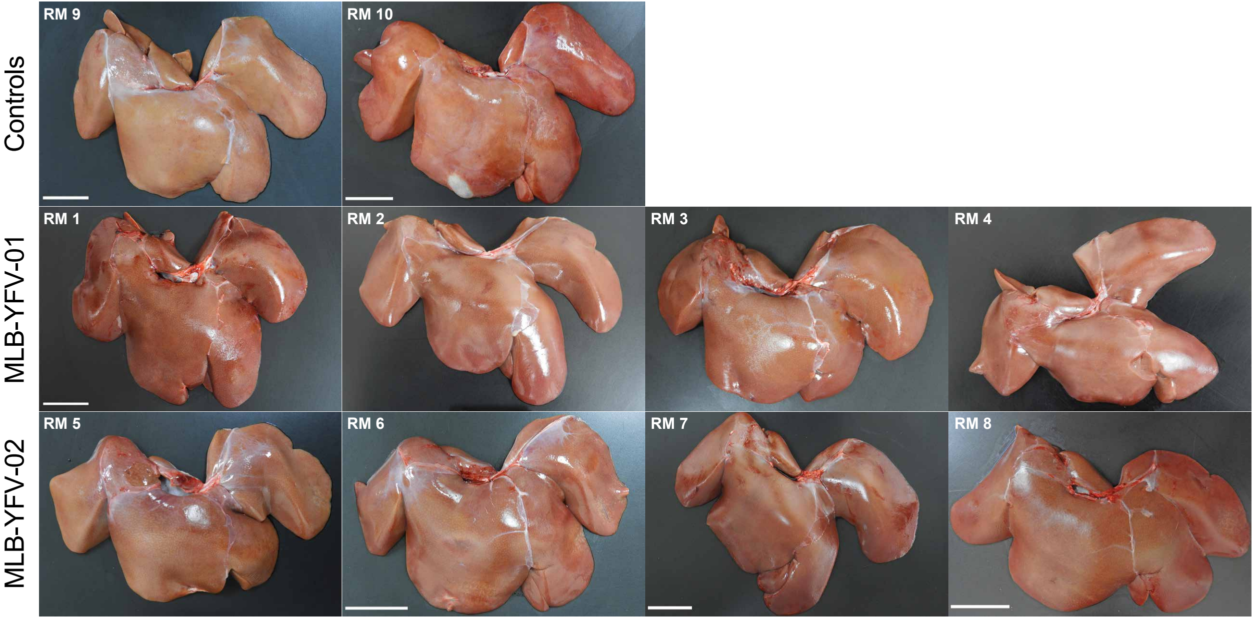

Figure S2. Gross pathology of livers of YFV-DakH1279 infected RMs. Livers were removed from euthanized RMs and immediately photographed. Bar = 3cm.

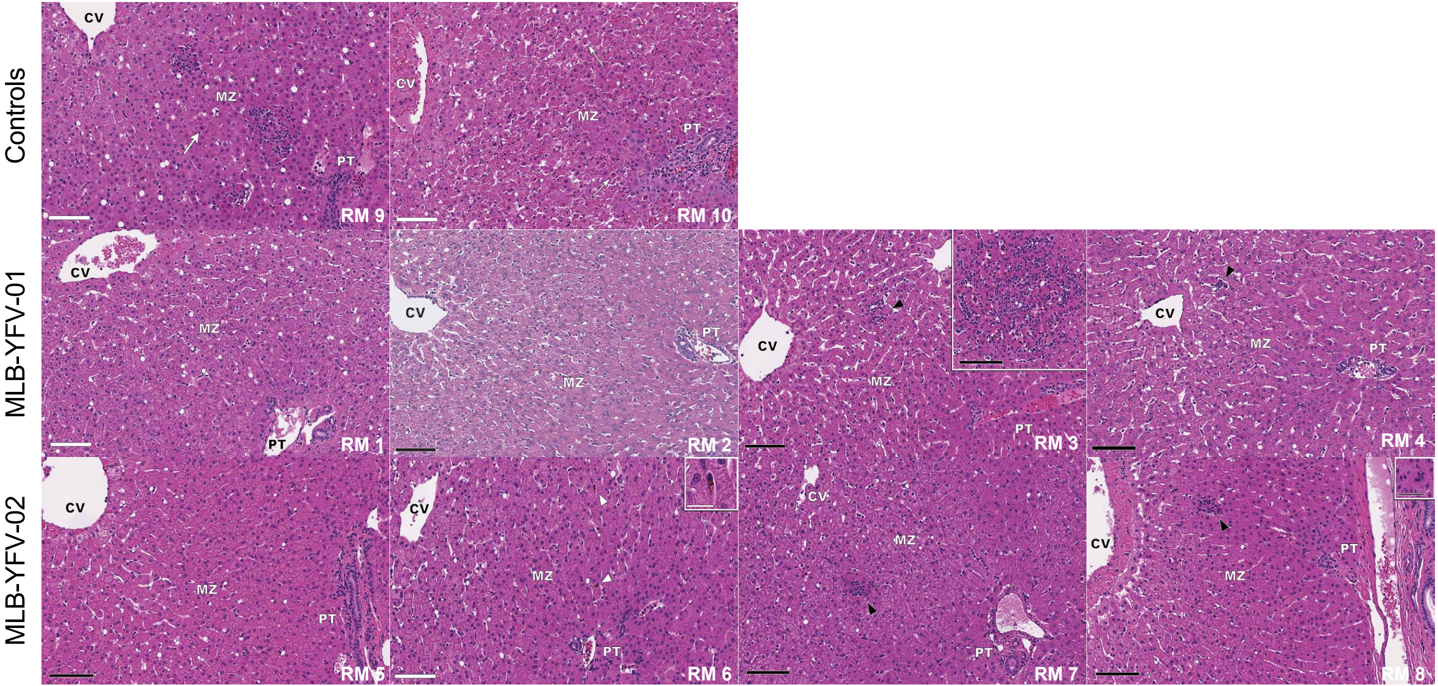

Figure S3. H&E staining from the livers of YFV-DakH1279 infected RMs. Normal histology from RM 1, 2 5, and 6 with rare midzonal Kupffer cells containing intracytoplasmic golden-tan pigment (white arrowhead, inset Bar = 25 μm) in RM 6. Minimal midzonal aggregates of lymphocytes (black arrowheads) in RM 3, RM 4, RM 7,and RM 8. 300 μm midzonal focus of necrotic hepatocytes surrounded and infiltrated by mononuclear inflammatory cells from RM 3 (Inset bar = 100 μm). Multinucleated hepatocyte in RM 8 (inset, Bar = 50 μm). PT = Portal triad, MZ = Midzonal region, CV = Central vein.

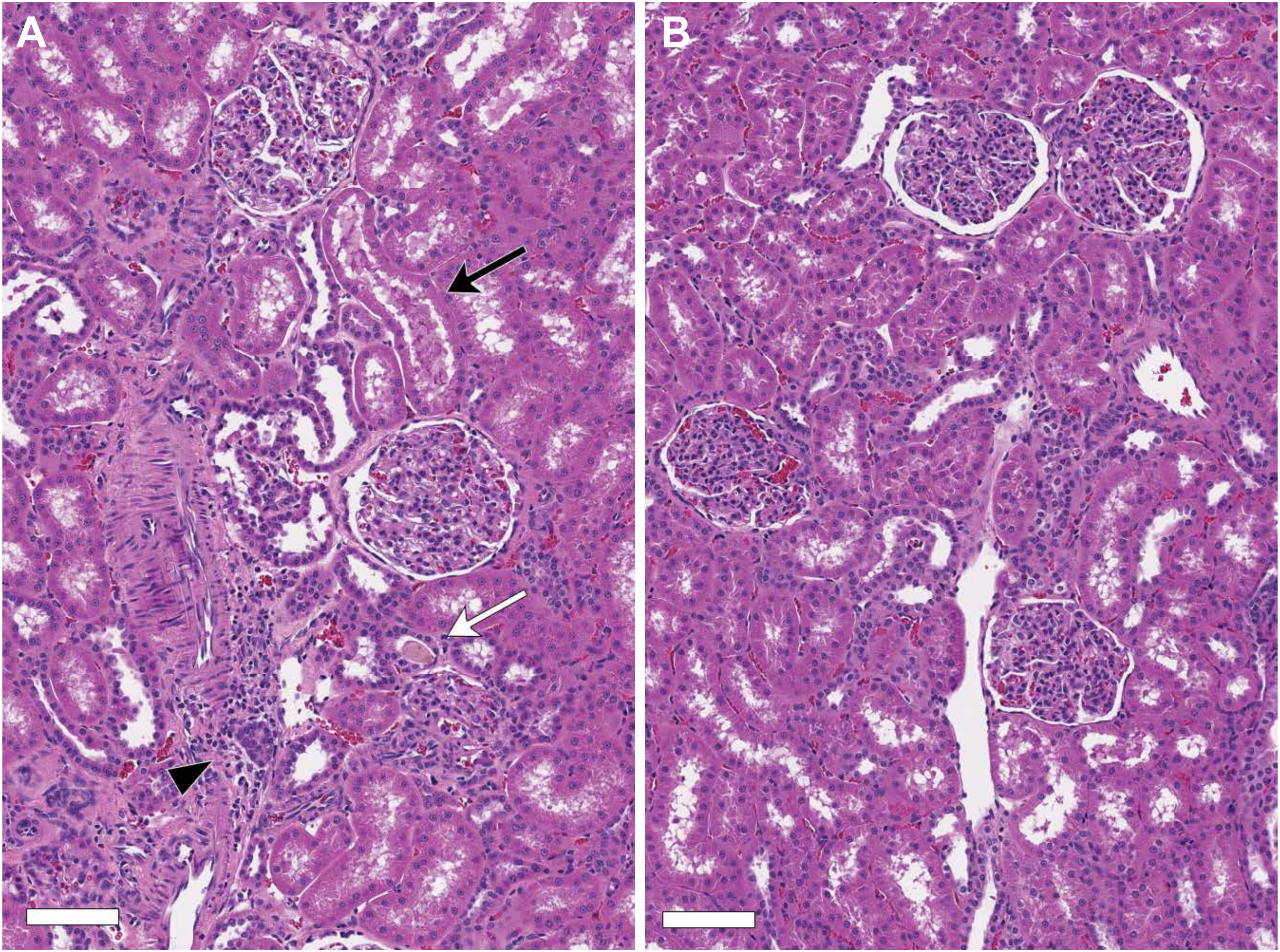

Figure S4. H&E staining from the kidneys of YFV-DakH1279 infected RMs. (A) Tubular epithelial cell degeneration and necrosis, tubular ectasia with cellular, proteinaceous (black arrow), and bile (white arrow) casts with multifocal mild perivascular (black arrowhead) and interstitial lymphocytic inflammation from control RM 9. (B) Normal renal histology from treated RM 6. Bar = 100 µm

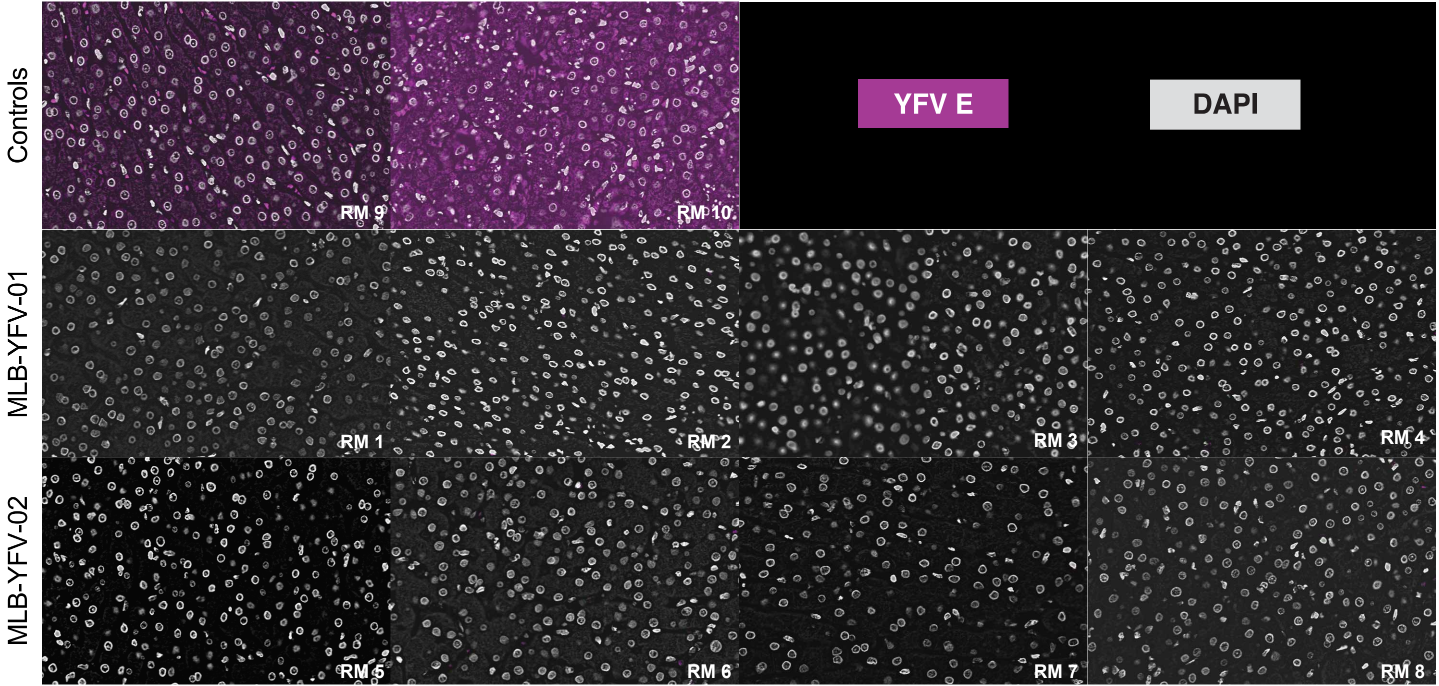

Figure S5. Immunofluorescent staining of YFV-DakH1279 E antigen in the livers of YFV-DakH1279 infected RMs. Note: use of E protein-reactive antibodies to stain virus-infected cells may overestimate the number of infected cells because it can detect virus particles attached to the surface membrane.

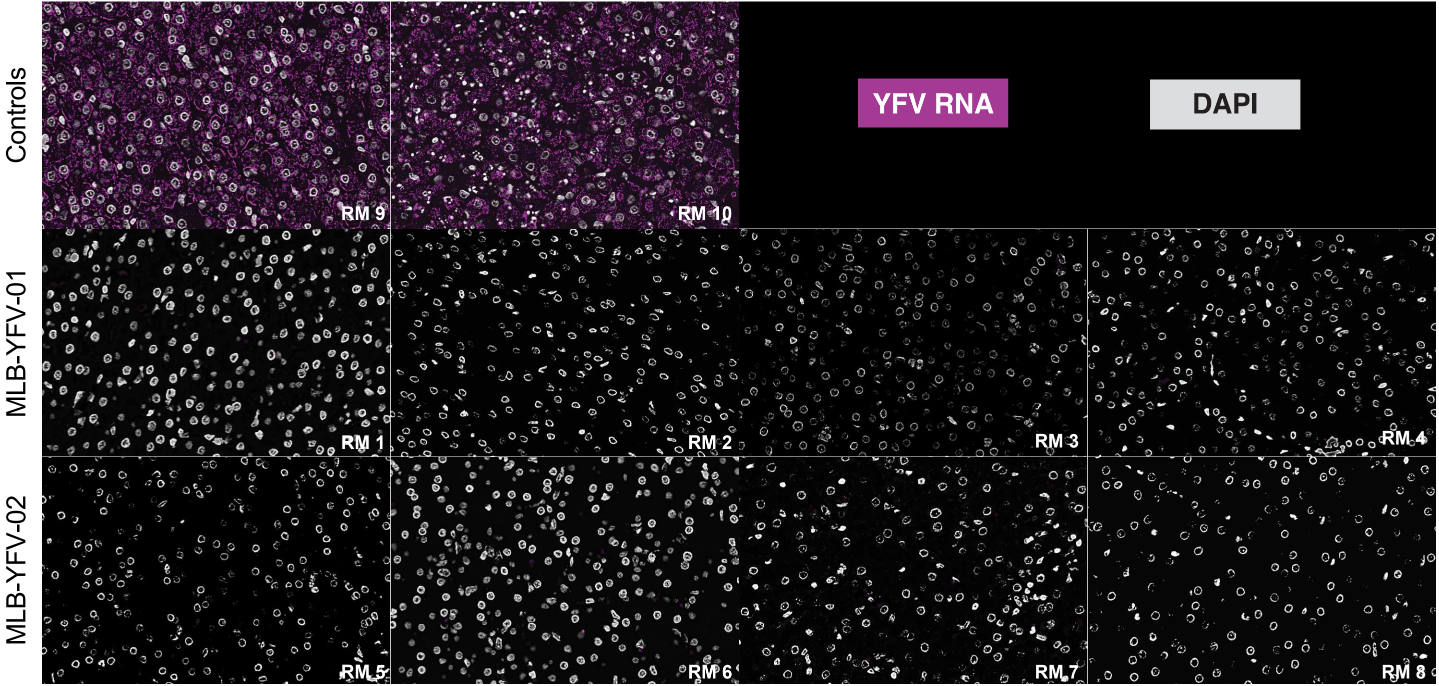

Figure S6. RNAScope staining of YFV-DakH1279 RNA in the livers of YFV-DakH1279 infected RMs. Liver sections were stained with RNA probes specific to YFV and imaged on an Olympus VS120 Slide Scanner.

mAb	mAb Concentration (µg/mL)	Passage Number									
		1	2	3	4	5	6	7	8	9	10
MBL-YFV-01	2	n.c.	n.c.	n.c.	n.c.	n.c.	n.c.	n.c.	n.c.	n.c.	n.c.
MBL-YFV-01	1	n.c.	n.c.	n.c.	n.c.	n.c.	n.c.	n.c.	n.c.	n.c.	n.c.
MBL-YFV-01	0.5	n.c.	n.c.	n.c.	n.c.	n.c.	n.c.	n.c.	n.c.	n.c.	n.c.
MBL-YFV-02	2	n.c.	n.c.	n.c.	n.c.	n.c.	n.c.	n.c.	n.c.	n.c.	n.c.
MBL-YFV-02	1	n.c.	n.c.	n.c.	n.c.	n.c.	n.c.	n.c.	n.c.	n.c.	n.c.
MBL-YFV-02	0.5	n.c.	n.c.	n.c.	n.c.	n.c.	n.c.	n.c.	n.c.	n.c.	n.c.
MBL-YFV-03	2	n.c.	n.c.	n.c.	n.c.	n.c.	n.c.	n.c.	n.c.	n.c.	n.c.
MBL-YFV-03	1	n.c.	n.c.	n.c.	n.c.	n.c.	n.c.	n.c.	n.c.	n.c.	n.c.
MBL-YFV-03	0.5	n.c.	n.c.	n.c.	n.c.	n.c.	n.c.	n.c.	n.c.	n.c.	n.c.
MBL-YFV-04	2	n.c.	n.c.	H67R	H67R	H67R	H67R	H67R	H67R	H67R	H67R
MBL-YFV-04	1	n.c.	n.c.	n.c.	n.c.	I70T + A147V	I70T + A147V	I70T + A147V	I70T + A147V	I70T + A147V	I70T + A147V
MBL-YFV-04	0.5	n.c.	n.c.	n.c.	n.c.	n.c.	n.c.	S325L	S325L	S325L	S325L
MBL-YFV-05	2	n.c.	n.c.	n.c.	n.c.	n.c.	n.c.	n.c.	n.c.	n.c.	n.c.
MBL-YFV-05	1	n.c.	n.c.	n.c.	n.c.	n.c.	n.c.	n.c.	n.c.	n.c.	n.c.
MBL-YFV-05	0.5	n.c.	n.c.	n.c.	n.c.	n.c.	n.c.	n.c.	n.c.	n.c.	n.c.
None	-	n.c.	n.c.	n.c.	n.c.	n.c.	n.c.	n.c.	n.c.	n.c.	n.c.

Table S1. YFV-17D escape mutations following 10 passages in vitro under mAb selective pressure. n.c. = No amino acid change detected from orignal infecting YFV-17D virus sequence.

Days post-infection	Hamster weight (Fig. 2C)	RM viral loads (Fig. 3C)
0	1.0000	1.0000
2	-	0.9000
3	0.9992	<0.0001
4	1.0000	<0.0001
5	0.6578	<0.0001
6	0.0015	-
7	0.0002	-
10	<0.0001	-
14	<0.0001	-
21	-	-

Table S2. Two-way repeated measures ANOVA analyses (p-values).

Animal	Sex	Age (days)	Weight (kg)	Origin	MHC (Mamu-)
RM 1	M	1,709	8.0	India	A1*004, A1*008, A2*05, A3*13, A4*14, B*012, B*022, B*030, B*031, B*057, B*074, B*082, B*098
RM 2	M	1,422	5.6	India	A1*004, A4*14, B*001, B*030, B*049, B*057, B*060, B*072, B*097
RM 3	M	1,352	7.3	India	A1*008, A1*025, A2*05, A3*13, A4*14, B*005, B*006, B*008, B*015, B*044, B*072, B*079
RM 4	M	1,352	5.8	India	A1*008, A1*012, A2*05, A3*13, B*041, B*048, B*052, B*055, B*058, B*064
RM 5	F	2,121	5.5	India	A1*001, A1*004, A2*05, A4*14, B*001, B*017, B*029, B*030, B*051, B*061, B*063, B*068, B*072
RM 6	M	1,353	5.0	India	A1*002, A1*023, A3*13, A4*14, B*001, B*012, B*030, B*057, B*082, B*098
RM 7	M	1,346	5.7	India	A1*001, A1*004, A2*05, A4*14, B*002, B*005, B*015, B*072, B*079, B*098
RM 8	M	1,330	5.2	India	A1*019, A1*023, A2*05, A4*14, B*021, B*028, B*046, B*051, B*056, B*067, B*068, B*072, B*079, B*082, B*124
RM 9	M	3,168	11.3	India	A1*002, A1*004, A3*13, A4*14, B*012, B*030, B*046, B*048, B*053, B*057, B*072, B*082
RM 10	M	1,748	10.3	India	A1*004, A1*059, A4*14, A7*02, B*012, B*017, B*022, B*029, B*031, B*057, B*060, B*061, B*068, B*098

Table S3. Demographics of RM utilized in YFV challenge study. MHC = major histocompatibility complex. Mamu = Macaca mulatta.

Animal ID	Pathology reported
RM 1	Epistaxis<br>Alopecia, marked, occipital region, limbs, flanks<br>Adhesion, focal, chronic, minimal, left caudal lung lobe and diaphragm
RM 2	Excellent physical condition<br>No gross lesions
RM 3	No gross lesions
RM 4	Excellent physical condition<br>No gross lesions
RM 5	Hypertrophic cardiomyopathy, chronic, moderate<br>Hyperemia, fundus<br>Alopecia, multifocal, minor
RM 6	Alopecia, multifocal, mild
RM 7	Enteritis, diffuse, chronic, moderate, hyperplastic, jejunum<br>Dilatation, diffuse, moderate, middle mesenteric and jejunal serosal lymph vessels
RM 8	Lymphadenomegaly, multifocal, mild, peripheral and visceral lymph nodes
RM 9	Hepatic necrosis and steatosis, diffuse, acute, moderate to severe<br>Icterus, diffuse, minor, renal pelvic fat<br>Dental calculus, multifocal, chronic, mild<br>Tension lipidosis, focal, chronic, mild, right medial liver lobe<br>Adhesions, multifocal, chronic, minimal, caudal lung lobes and diaphragm
RM 10	Hepatic necrosis and steatosis, diffuse, acute to subacute, severe<br>Dehydration, moderate<br>Splenomegaly, moderate<br>Icterus, multifocal, acute, mild, peripancreatic and renal pelvis fat<br>Lymphadenomegaly, multifocal, mild, peripancreatic lymph nodes<br>Dental calculus, multifocal, chronic, mild

Table S4. Gross NHP pathology reports.

Primer Name	Primer Sequence
YFV-M1-FWD	5’-actcagcaggcaggtctag-3’
YFV-E1-FWD	5’-gagcaagacaagtgtgtcac-3’
YFV-E2-FWD	5’-gtttgaggttgatcagacc-3’
YFV-E3-FWD	5’-aaaccaggaaggctccttg-3’
YFV-E4-FWD	5’-ctgattgaggtgaacccac-3’
YFV-M1-REV	5’-cttcgttctcttcagctagg-3’
YFV-E1-REV	5’-ctgtccactatccagctctc-3’
YFV-E2-REV	5’-gtgtcagttgggttcttgac-3’
YFV-E3-REV	5’-tccctctttgtgccactg-3’
YFV-E4-REV	5’-gaagataccatctccgcac-3’

Table S5. YFV-E-specific primers used for Sanger sequencing.
